# “Every shoulder is different”: A qualitative study of clinicians’ insights on the causative factors and strategies of managing work-related shoulder disorders among firefighters

**DOI:** 10.1371/journal.pone.0348934

**Published:** 2026-05-21

**Authors:** Temitope A. Osifeso, Joy C. MacDermid, Gojjam Limenih, Mike Szekeres, Pulak Parikh, Kenneth J. Faber

**Affiliations:** 1 Department of Health and Rehabilitation Sciences, Western University, London, Ontario, Canada; 2 Clinical Research Lab, Roth McFarlane Hand and Upper Limb Centre, London, Ontario, Canada; 3 Lawson Health Research Institute, London, Ontario, Canada; 4 Department of Surgery, Western University, London, Ontario, Canada; Universiti Kebangsaan Malaysia, MALAYSIA

## Abstract

**Background:**

Work-related shoulder disorders (WSDs) are the third most prevalent work-related musculoskeletal disorder in firefighting, a significant occupational health concern among firefighters (FFs). However, comprehensive understanding of clinician perspectives on causative factors and evidence-based management strategies remains limited.

**Objective:**

To explore clinicians’ perspectives on: (a) the underlying causative factors contributing to WSDs; and (b) management strategies specific to firefighters.

**Methods:**

Semi-structured interviews were conducted with 15 clinicians (11 males, 4 females) between 30–61 years of age, who had experience managing firefighters with WSDs. Data collection and analysis followed interpretive description methodology, employing iterative thematic analysis to identify recurring themes and patterns.

**Results:**

Eight themes emerged: 1) The Nature of Firefighting Work Exacerbates Shoulder Injuries; 2) Work-Related Slips and Falls are Linked to Traumatic Shoulder Injuries; 3) Unequal Shoulder Injury Pattern by Sex and Work Experience; 4) Lack of Formal and Targeted Training Exercises Predisposes to Shoulder Injuries; 5) Early Diagnosis is Crucial for Effective Shoulder Management; 6) Individualized Multimodal Treatment Approaches are Key to Optimal Recovery; 7) (Re-)Education on Safe Exercise Training Minimizes Shoulder (Re-)Injuries; 8) Specialized functional Assessment and Outcome Measures could Enhance Treatment Outcomes.

**Conclusion:**

Firefighter WSDs are multifactorial, exacerbated by occupational exposures, sociodemographic factors and delayed or non-specialized care. Clinicians emphasize the need for implementing targeted functional rehabilitation approaches that consider the unique physical and operational demands of firefighting duties.

## Introduction

Firefighting is a physically demanding occupation, characterized by unpredictable emergency responses, hazardous environmental exposures, and intense physical exertions [1]. The occupational demands inherent in firefighting operations, including victim rescue, fire suppression, and repetitively maneuvering heavy equipment overhead increases the risk for work-related shoulder disorders (WSDs) [2,3]. Several studies indicate that WSDs constitute the third most prevalent work-related musculoskeletal disorder (WRMSDs) among firefighters (FFs), following only lower back and knee injuries [4,5]. For instance, a systematic review by Tahernejad et al., [6] showed that WSDs represent approximately 18% of WRMSDs reported by firefighters.

Likewise, emerging studies have emphasized the unique challenges female firefighters face in relation to equipment fit [7], physiological differences, and workplace cultural factors [8] and how that may influence both shoulder injury risk and treatment outcomes [2]. Understanding how these factors interact with occupational injury mechanisms and treatment approaches is essential for developing a comprehensive and equitable management approach.

The unique occupational demands of firefighting necessitate specialized clinical understanding of WSDs that account for both the acute traumatic mechanisms such as falls from heights or equipment-related injuries and the chronic overuse patterns including repetitive overhead tasks peculiar to the profession [9]. For example, a study by Killip et al., [10] noted a lack of tailored management protocols for firefighters with WRMSDs including WSDs. Hence, the study emphasized the importance of utilizing firefighter-specific management protocols by clinicians who have experience working with firefighters.

Studies have also shown that WRMSD like WSDs, compromise firefighter health and career sustainability, imposing a significant operational and economic burden on fire departments and the healthcare systems [11,12]. For instance, a Canadian study by Frost et al., [13] reported that the combined medical and compensation cost of WSDs among firefighters in 2012 was about 76000CAD per annum. Despite the significant cost, high prevalence and negative impact of shoulder disorders in firefighting populations, limited research has specifically examined clinician perspectives on the causative mechanisms and optimal management approaches for WSDs.

While most firefighter-focused research has identified general musculoskeletal disorder patterns [5,14,15], there remains a significant gap in understanding how healthcare professionals who specialize in treating firefighting personnel conceptualize shoulder injury causation and structure their treatment approaches to fit their intense occupational demands. The aim of this study is to explore clinicians’ perspectives on the: a) the underlying causative factors of WSDs among FFs; b) management strategies specific to FFs with WSDs. Through the exploration of healthcare professional experiences and clinical reasoning. Understanding these clinical perspectives is crucial for developing targeted interventions that address the multifaceted nature of WSDs among firefighters.

## Methodology

### Study design

Interpretive Description (ID) was adopted as the methodological framework for this study due to its strong alignment with the goals of applied clinical research and its capacity to generate practice-informed insights [16]. Specifically, ID was developed to bridge the gap between academic inquiry and healthcare contexts, offering a pragmatic and theoretically flexible approach to exploring complex experiential questions [16]. It also accounts for the uniqueness of individual narratives and the patterns of shared experience that emerge across participants [17,18]. In our study, it allowed us to meaningfully examine the lived experiences of clinicians who have managed FFs with WSDs, focusing on the common causative factors of WSDs and how they have treated them in their practices. Interpretive description is rooted in the constructivist paradigm and recognizes that knowledge is co-constructed through dialogue between the researcher and participants. This relational stance proved essential in uncovering the nuanced ways clinicians make sense of firefighters’ WSDs and how they help re-integrate them back to their physically demanding roles. Hence, the ID methodology supports the development of interpretive insights that are both theoretically grounded and deeply relevant to the applied clinical decision-making and program development for this unique population.

### Statement of ethics approval

The study received ethics approval from the Western University Health Sciences Research Ethics Board (HSREB), Canada (Project ID#122676), and was conducted in full compliance with the ethical principles outlined in the Declaration of Helsinki [19].

### Sampling, recruitment, and consent

A purposeful sampling approach was used to recruit clinicians, including physiotherapists and orthopedic surgeons who had at least one year of experience and have also managed WSDs among FFs across Canada. Sampling aimed to capture variation in geographic regions, years of practice, and healthcare settings (e.g., public and private institutions) to ensure a rich understanding of how WSDs are referred, diagnosed, and treated within various clinical environments. Recruitment took place between October 30, 2023, to July 10, 2025, through professional networks like clinician referrals (word of mouth) in various private and public clinical settings and professional organizations. Clinicians were provided with detailed letters of information, outlining the purpose of the study, procedures, and voluntary nature of participation. Verbal and written informed consent were obtained prior to the start of each interview and clinicians were given a 25 CAD Amazon gift card after the interviews. To maintain participant anonymity, no identifiable demographic or institutional details were linked to the data, and each clinician was assigned a unique identification number in the order they were interviewed. Effort was made to ensure representation across sex, gender, years of clinical experience, and practice settings, recognizing that such factors could influence perspectives on care delivery, decision-making, and systemic barriers. For instance, through referrals, we sent direct emails to female orthopedic surgeons as we noticed that we had only male participants in our study. Attention to these dimensions enriched the interpretive depth of the study, particularly as they relate to clinician reasoning and institutional constraints. Sample adequacy was evaluated iteratively during data collection and recruitment was concluded once sufficient experiential variation and thematic richness were achieved to meet the study analytical and practical objectives [20].

### Data collection

Data was collected by the first author (TO) through semi-structured interviews using a flexible, participant-centered approach. Interviews were conducted primarily via Zoom and participants were also given the option to choose alternative formats, including telephone or in-person, to support accessibility. Prior to each session, verbal and written informed consent was obtained and the rights of participants were protected. Interviews ranged from approximately 30 minutes to one hour in length. The interview guides (see S1 and S2 Files) were developed iteratively, grounded in the research team’s applied clinical knowledge, experience with firefighter health, and an appraisal of existing literature. Using open-ended questions, the interview guides (see S1 and S2 Files) helped to drive the conversation, allowing for consistent inquiry while giving participants opportunities to elaborate on their lived experiences and management strategies. All interviews were conducted and transcribed verbatim by the first author (TO). Field notes were also documented immediately to capture contextual nuances, emotional tone, and preliminary analytic impressions. The guide was refined iteratively throughout the study, with insights from earlier interviews informing subsequent conversations, which are consistent with ID approach of analytical responsiveness and inductive inquiry. This process allowed for the co-construction of meaning between researchers and participants, enriching the data’s interpretive depth and grounding it in the realities of clinical practice.

### Data analysis

Data analysis was guided by the principles of ID, with an emphasis on contextual depth, applied relevance, and reflexive engagement throughout the analytic process. Interview transcripts were anonymized and imported into NVivo 15 to support transparent data management, facilitate coding, and enable systematic retrieval. Analysis was initiated concurrently with data collection, consistent with the iterative nature of ID. Drawing on Braun and Clarke’s six-phase framework for thematic analysis [21], this approach supported the emergence of thematic patterns while staying grounded in the clinical perspectives shared by participants. The first author (TO) transcribed each interview and immersed herself in the data through repeated readings. To identify patterns of meaning, a pilot coding exercise was conducted independently by two co-authors (TO and GL) on a selected transcript to establish analytic alignment and coding consistency. Discrepancies were later discussed and resolved collaboratively during another one-on-one meeting. Subsequently, the remaining transcripts were coded independently by the two researchers (TO and GL), with periodic analytic meetings to discuss emerging interpretations and refine the thematic structure. A coding tree was developed iteratively, beginning with initial descriptive codes derived from the transcripts. These codes were grouped into categories, which were then refined into overarching themes and sub-themes. Reflexivity was embedded throughout the process. Memos and field notes were used to document analytic decisions, and to critically examine personal, professional, and epistemological positioning. These strategies ensured transparency and contributed to the rigor of the analysis. While the study was originally designed to explore the treatment strategies employed by clinicians when managing FFs with WSDs, comprehensive analysis identified common contributing factors that influence WSDs among firefighters and the various management strategies clinicians employed. These findings prompted the research team to broaden the analytical framework to examine both intervention approaches and the underlying causative factors of firefighter WSDs.

### Researcher positionality

As a female doctoral researcher, my background in physiotherapy offered a grounded appreciation for the biomechanical and rehabilitative complexities of musculoskeletal injuries, while my prior engagement with firefighter health research sensitized me to the occupational demands, injury mechanisms, and recovery challenges unique to them. Recognizing that clinicians including orthopedic surgeons and physiotherapists are the informants of this lived experience, I approached the research with a deep respect for the clinical reasoning, professional values, and systemic constraints that shape healthcare delivery. As someone outside the surgical profession but familiar with rehabilitative pathways, I remained aware of the boundaries and bridges between disciplinary standpoints. Rather than striving for neutrality, I acknowledged my interpretive role within a constructivist paradigm, where meaning is co-constructed through dialogue. Throughout the process, I employed reflexive strategies such as memoing and critical journaling to interrogate how my assumptions, prior knowledge, and discipline training could influence what I noticed, questioned, and represented. In engaging with orthopedic surgeons and physiotherapists, I was mindful of power asymmetries in the clinical hierarchies discussed and treated their narratives as situated insights shaped by systematic pressures and clinical judgment. My responsibility as a researcher grounded in ID was to translate these accounts into meaningful patterns and actionable knowledge without distorting the integrity of what was discussed. Ultimately, my positionality in this study employed relational awareness and methodological transparency by remaining mindful of the tensions and contradictions of clinician perspectives and being reflexively engaged in the co-construction of meaning to ensure a clinically relevant finding to improve the care of FFs with WSDs.

### Rigor and trustworthiness

To uphold the trustworthiness of this study, we employed multiple, integrated strategies that reflect the interpretive descriptive nature of our methodology and the clinical relevance of the topic. Credibility was established through sustained engagement with participants, which allowed for the development of rich accounts of their experiences managing FFs with WSDs. We prioritized depth by probing into the practical, ethical, and systemic dimensions of the clinicians’ clinical decision-making. Transparency was ensured through the maintenance of a detailed audit trail, documenting analytical pathways, coding iterations, and interpretive turning points. To ensure transferability, we provided thick, contextualized descriptions that situate participants’ narratives within the broader realities of clinical care and occupational health. These descriptions were done to ensure the relevance and applicability of our findings to other high-risk occupations or musculoskeletal-related settings. Reflexivity was actively practiced throughout the study as a continuous, iterative process. Through memo writing and critical dialogue, we examined how our disciplinary training, prior assumptions, and clinical affiliations shaped the analytic lens. Our goal was not to eliminate bias but to make visible the interpretive process and ensure that emergent themes reflected participants’ lived realities rather than our own preconceptions or bias. Finally, we engaged in analytic triangulation through peer debriefing. Specifically, senior colleagues with expertise in musculoskeletal rehabilitation, mental and occupational health of high-risk population like FFs reviewed segments of the data and thematic interpretations. Their feedback helped refine analytical clarity and reduce the risk of interpretive bias, strengthening the overall coherence and integrity of our findings.

## Findings

### Participants’ characteristics

We recruited and conducted interviews with 15 clinicians (men = 11, women = 4) from different geographical locations across Canada, including 14 from Central Canada (i.e., Ontario, Quebec), 1 from Atlantic Canada (i.e., Nova Scotia), and 1 from Western Canada (i.e., British Columbia). *See* Table 1. Participants were all interviewed virtually through Zoom, and we had an equal number of physiotherapists (n = 8) and orthopedic surgeons (n = 8). The clinicians were mostly Caucasians (n = 10) with an age range between 30–61 years of age. The most reported WSDs among clinicians managing firefighters were rotator cuff tears (RCTs) and shoulder instability. See Table 1.

**Table 1:** Clinicians’ demographics.

ID	Sex	Gender	Age	Ethnicity	Type of WSD Managed	Years of Experience	Province
PT01	Male	Man	45	South Asian, Indian	RCTs, Shoulder arthritis, Labral Pathology	21	Ontario
PT02	Male	Man	43	West African, Nigerian	RCTs, Scapular Dyskinesia, Labral Injuries, Shoulder Dislocations	22	Ontario
PT03	Female	Woman	61	Caucasian	RCTs, Bicep Tendinopathy, Anterior Shoulder Instability & Shoulder Dislocations	18	Nova Scotia
PT04	Male	Man	46	Caucasian	Subacromial impingement, Frozen Shoulder (Adhesive Capsulitis), Anterior Shoulder Instability	22	Ontario
PT05	Female	Woman	39	Caucasian	RCT (supraspinatus tear), Bicep Tendinopathy	19	Quebec
PT06	Female	Woman	37	Caucasian	RCTs and Rotator Cuff Tendinopathy	15	Quebec
PT07	Male	Man	30	Caucasian	AC separation, Supraspinatus impingement and Biceps Tendinopathies	1.5	British Columbia
OS08	Male	Man	61	Caucasian	RCTs, Bicep Tendinopathy	35	Ontario
OS09	Male	Man	60	Caucasian	RCTs, Arthritis	34	Ontario
PT10	Male	Man	33	West Asian – Iranian	RCTs	10	Ontario
OS11	Male	Man	51	Caucasian	RCTs, Labral Tears, Shoulder Instability	25	Ontario
OS12	Male	Man	37	South-Asian- Indian	RCTs, shoulder instability, labral tears, Arthritis.	5	Ontario
OS13	Male	Man	53	Caucasian	Posterior Shoulder Dislocation	22	Ontario
OS14	Male	Man	54	Middle East-Arab	RCTs, Shoulder instability and then Arthritis	25	Ontario
OS15	Female	Woman	54	Asian	RCTs	18	Ontario

OS: Orthopedic surgeon; PT: Physiotherapist; RCTs: Rotator cuff tears; AC: Acromioclavicular separation

## Major themes

**Fig 1:**
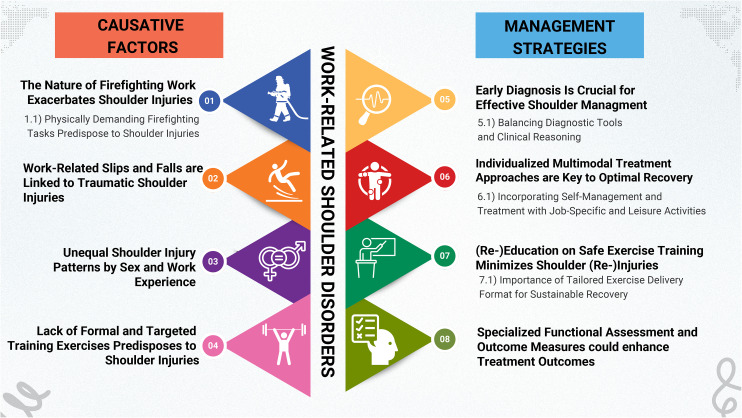
Conceptual model of clinicians’ perspective of WSDs among firefighters.

Through rigorous coding and thematic analysis of clinicians’ interviews, seven major themes and five sub-themes emerged, as shown in Fig 1. These themes showed the underlying causative factors encountered by clinicians during their practice and the complex interplay of interprofessional collaboration in managing WSDs among FFs.

### Causes of work-related shoulder disorders among firefighters

#### Theme 1: The nature of firefighting work exacerbates shoulder injuries.

All clinicians stressed that FFs experience earlier onset of shoulder pathologies compared to the general population, suggesting occupational hazards as a contributing factor. One of the clinicians stated:

*“They’re getting disease earlier than others… I think there is an occupational element to why they’re presenting with cuff disease earlier…”* ***OS08-Male.***

The other clinician added that the nature of firefighting tasks can be largely unmodifiable, thus posing challenges to implementing primary prevention without compromising their operational tasks.

*“I don’t know how that (shoulder injury) could change... it’s such an essential job with intense physical demand.”* ***PT05-Female.***

They further noted that their deep sense of duty and work culture, although admirable, can have a negative impact in terms of delayed reporting when they experience an injury which can lead to further complexity when they finally seek medical care.

*“They are aware(of shoulder injury)… but in a bit of denial… until they start having issues. It’s really hard to help somebody else if you haven’t helped yourself first.”* ***PT07-Male***

Furthermore, some clinicians reported that adverse scheduling and shift work of firefighter organizations often led to fatigue of FFs, increasing their risk for WSDs.

“*Extended hours... overtime... unpredictable workplace combined with fatigue may put them (firefighters) at higher risk of (shoulder) injury.”****PT04-Male*.**

Despite these intense shift schedules, clinicians stated most FFs are willing to “sacrifice their bodies” for long period of time which can often result in more severity especially with degenerative changes of the shoulder.

*“…I think there’s a psychological element there…they like to rescue people and they like to think of themselves as heroic in some way, whether they acknowledge it or not, …But I think because of that, they will sacrifice their bodies a little bit longer than the average person.”* ***PT03-Female***

#### Sub-Theme 1.1: Physically demanding firefighting tasks predispose to shoulder injuries.

Most clinicians agreed that firefighting often requires some unpredictable tasks or roles that predispose them to bodily injuries, including WSDs. They indicated that FFs are required to carry out most of their tasks in an unpredictable work environment and sometimes in very dangerous situations predisposing them to shoulder injuries. One of the clinicians stressed that:

*“Firefighters are limited in their ability to plan out their roles, sometimes because they may not know what’s happening until they get on site…So, the predictability in their work site is low for those in the field and it’s more of a matter of the preparation of their body to be able to handle who knows what.”* ***PT04-Male.***

Further, clinicians mentioned that firefighting tasks and the expectations of the job mostly require the use of their shoulders repetitively, in awkward positions and mostly above shoulder height while maneuvering heavy equipment. These continuous work-related tasks were claimed to result in various types of degenerative shoulder conditions including the early onset of arthritis and rotator cuff tears.

*“I think that when you think about it, they are doing a lot of work with their arms and over your head stuff when you’re looking at, …their work activities or work duties, there is some repetitive movement overhead movement that’s considerable.”* ***PT01-Male.***

One clinician emphasized that firefighting tasks such as lifting, and overhead activities were identified as major contributors to shoulder injuries.

*“I think maybe the overhead activities and also pulling and lifting activities that they have in their job would be one of the most causes of the pain and (shoulder) problems.”* ***PT10-Male.***

Another clinician noted that physically demanding roles can be life altering for FFs causing life threatening circumstances while they are carrying out their duties.

*“…I feel like someone who has a less physically demanding job, you could probably, you know, stop at about 50 to 75% of the way and you know they’ll safely get on with the rest of their lives versus these patients that really have to be fully functional and you know it’s a life or death situation..”* ***PT05-Female***

### Theme 2: Work-related slips and falls are linked to traumatic shoulder injuries

Clinicians reported that another critical cause of traumatic shoulder injuries among FFs are slips and falls often resulting from high impact physical activities at work. They mentioned that slips and falls often place the shoulders in vulnerable positions that predispose it to dislocations, significant rotator cuff tears, and acromioclavicular joint subluxation.

*“The major one that I would say is the ambulation over uneven terrain, especially in forest firefighting. Two of the injuries I’ve had came from slip and fall like the subluxation came from slipping while moving over a tree and pushed into the crank position and dislocating on them. And then just moving over this uneven terrain into really like at a rapid pace because, it’s an emergency situation leading to them having falls that increase the risk of traumatic (shoulder) injuries...”* ***PT07-Male*.**

Likewise, these injuries were claimed to be commonly associated with falls from heights or ladders while carrying out their routine tasks at work and sometimes during training exercises among new recruits with less experience and skill

*“Well, I mean it’s a high-risk job where they are at risk of injuring their shoulders. The mechanism that I’ve come across has been falls, so at least with respect to the posterior shoulder dislocations,…I think I’ve had three firefighters that I can think of…So typically, the last one I treated just maybe six months ago, he was doing a simulation, a drill, and he fell from the fire tower and he dislocated his shoulder posteriorly…”* ***OS13-Male.***

In addition, a physiotherapist expressed that shoulder injuries might also be linked to biomechanical factors when trying to break a fall resulting in a traumatic shoulder injury

*“…but it’s again you having a traumatic experience at work, you know, something collapses on you. Right. And then you’re trying to break your fall and then you land have a fractured wrist, clavicle or even like a tear to the shoulder capsule. Those are some of the things that I’ve seen.”* ***PT02-Male***

#### Theme 3: Unequal shoulder injury patterns by sex and work experience.

Clinicians indicated that when considering shoulder injury patterns, the role of FFs at work and the length of service are important factors to consider as it gives some clarity on the cause of shoulder injury.

*“…not all firefighters have the same tasks or sort of occupational hazards. Some are kind of the chiefs and do less and supervise more and then others are more like, you know, the active duty individuals that are doing like the roles of whatever heavy labor tasks are involved, …but I assume they sort of take different roles at different times, so I usually get an idea of what their current role is and what tasks they’re asked to do on a regular basis, because oftentimes that’s one of the key indicators for me at least on why they might have an onset (of shoulder) pain, if they’ve been a firefighter for like 10 years, other times it’s just because of the fact that they’ve had enough time being a firefighter, for instance, like for more degenerative type things like rotator cuff tears and arthritis. In those patients, it’s often because they’ve been doing it for so long…”* ***OS12-Male.***

Clinicians often reported that chronic conditions such as arthritis and rotator cuff injuries were commonly experienced amongst older and more experienced FFs, while acute injuries can occur at any age or year of experience. Notably, a clinician said:

“*Most of the people that I see would be somewhat experienced. Chronic (shoulder) injuries happen with age... acute injuries... age is less of a factor…”* ***OS09-Male.***

Further, two clinicians asserted that they managed a predominantly male patient base compared to female FFs and this may be attributed to the nature of the job, physical demands and male-domination of firefighting.

“*. The vast majority I have managed are male, I think it’s just the nature of it. It’s a job that appeals to males, it’s physically demanding, many females may not be interested in doing...”* ***OS09-Male.*** *“…From all the cases that I can recall have been male. I don’t remember treating any female firefighters…They’re overwhelmingly male, yeah.”* ***OS13-Male.***

#### Theme 4: Lack of formal and targeted training exercises predisposes to shoulder injuries.

Clinicians expressed concern that most firefighters over-train and use inappropriate exercise techniques in the gyms which increase their risks for shoulder injuries.

*“So, I absolutely think that, you know, kind of double dipping in that, you know, overuse category for sure is that they’re over working in the gyms and then some of the gyms aren’t that great, like, they’re not good quality. So, they’re kind of potentially, you know creating (shoulder)injuries in that...”* ***PT01-Male.***

Another clinician expressed similar opinion that most FFs lack certain exercise training needed to carry out certain firefighting duties and that most exercise routines do not simulate their firefighting routines.

*“…I think firefighters end up carrying a lot on their shoulders. They also do a lot of climbing, if you think about climbing the ladder up and down with things on them or over their shoulder and like just holding a pressured fire hose for long periods of time. It requires a lot of endurance. And so, I think they aren’t always well trained for that, particularly volunteer firefighters, which is most of what my population is.”* ***PT03-Female.***

This was supported by another clinician who noted the lack of ongoing formal exercise training program during their active duty rather than only at the beginning of their career. This was deemed important by clinicians to ensure sustainable conditioning throughout their firefighting career

*“…So, I think that, you know, I do agree that having some kind of formal recommendation for training is key, like as they are training and becoming firefighters, they have a training program. But I think once they’re firefighters. There’s nothing to maintain that and I think that doing something to optimize their strength and conditioning would probably improve their kind of workplace related (shoulder) injuries or at least reduce them, so putting together some kind of exercise program that kind of focuses on periscapular, rotator cuff strengthening and scapular stabilization, that’s kind of maintained throughout the course of their careers…”* ***OS12-Male***

### Management strategies for work-related shoulder disorders

#### Theme 5: Early diagnosis is crucial for effective shoulder management.

Clinicians emphasized that early detection and diagnosis are crucial not only for clinical outcomes but also to meet firefighters’ need for clarity. They indicated that most FFs are highly motivated and eager to understand their condition including the timeline for recovery.

*“Early diagnosis and intervention is important, especially because if we’re dealing with a subluxation, there’s so many structures that are surrounding that shoulder that it’s important that it gets relocated very quickly and done under monitoring… otherwise it’s going to be a prolonged recovery.”* ***PT07-Male.***

Notably, clinicians stated that psychosocial stressors and trauma histories can complicate assessment, suggesting the need for a trauma-informed lens in clinical encounters.

*“They want to know what’s going on... diagnosis detection should be at the forefront. There is mental health that goes on with that... psychosocial drivers... hidden neck pain that gets disguised as shoulder pathology.”* ***PT01-Male.***

Further, they mentioned that FFs often continue working despite their shoulder injuries, increasing the risk of chronicity and that early detection is essential to prevent minor shoulder injuries from escalating into complex disorders.

*“Yes, I think the time sensitive diagnosis in the shoulder tends to be the rotator cuff tendon tears. So, knowing whether someone has full thickness, rotator cuff tear because often these are younger patients, if the tears are larger, say more than 2cm, that tends to involve an entire muscle, typically the supraspinatus and with those tears of that size, they retract quickly. They undergo atrophy and do develop some irreversible changes fairly quickly, so I think it’s very important to be kind of cognizant that it could be a full thickness tear and I would investigate and pursue treatment quicker than I might in I guess a more sedentary population.”* ***OS08-Male***

#### Sub-Theme 5.1: Balancing diagnostic tools and clinical reasoning.

There were varying opinions on arriving at the diagnosis of the type of WSDs among firefighters. Some clinicians preferred using a thorough clinical assessment including detailed history taking, special tests during assessments rather than diagnostic tools like magnetic resonance imaging (MRI) or ultrasounds,

*“… I think generally, I’ll just say most times the, you know, subjective history and objective tells you your diagnosis whether there’s a referral or not. But obviously when there’s an MRI and things of that nature, it helps you make your decision, but generally I think I’m pretty confident that we can make some of these diagnosis without it. If you can describe the mechanism of injury, then I think it’s easy to put things together from there and determine the diagnosis.”* ***PT02-Male.***

This was echoed by another clinician:

*“…I like, you know, my clinical exam better than an MRI or anything. Any sort of injury, I really like my clinical exam for their condition, because doing a good health history and that paired with kind of clinical kind of you know cluster of whether it’s range of motion, strength, special tests I think can really be useful to really help the diagnosis.”* ***PT01-Male.***

Other clinicians emphasized a biomechanical and functional diagnostic approach, with particular attention to scapular movement and positioning. While it was noted that no firefighter-specific diagnostic tools were used, most physiotherapists emphasized observational and palpatory methods (e.g., scapular realignment testing) to rapidly assess symptom response and function in relation to their occupational context.

*“I tend to look a lot at the relationship between scapular movements and their impact on shoulder pain. Thorough history taking... probing with regards to movements performed repetitively or tasks frequently performed.”* ***PT05-Female***

In contrast, other clinicians advocated for diagnostic tools such as MRIs, Ultrasounds, and X-rays in addition to a comprehensive physical examination. This was believed to be more robust especially when it concerns a high-risk occupation like firefighters.

*“I wouldn’t say my approach is different in terms of the expectations of the need of a diagnostic test, because there’s certain clinical practice guidelines that I rely upon based on research that would identify someone who has a full tear or is suspected of having a full tear. I should say that individual that regardless of role, a diagnostic ultrasound would be relevant. I would say in this patient population that the phrasing of my concerns towards the need of that investigation as it relates to their job and the potential need of them waiting for the results might be of a higher priority…”* ***PT04-Male.***

A clinician further expressed that balancing diagnostic tools and clinical assessments can also be dependent on the type of injury presented by the FFs.

*“Probably early assessment and physical examination is probably the most important, and then probably MRI would be the most important test, but not for everyone depending on the individual situation.”* ***OS11-Male.***

Another clinician mentioned combining a pharmacological approach to diagnostic tools to confirm diagnosis:

**“***…if they improved with a diagnostic subacromial injection, that pretty much confirms that it’s cuff related pain. And then from there, diagnostically, just from our healthcare system, I tend to do screening ultrasounds pretty quickly. So that’s to give me an idea of tearing or no tear. And if the tear looks like it’s a more complex pattern than I would push for an early MRI…”* ***OS09-Male***

#### Theme 6: Individualized multimodal treatment approaches are key to optimal recovery.

Clinicians reported various styles in the management of WSDs depending on their scope of practice, clinical skills, and presentation of the WSDs of the firefighter. Specifically, physiotherapists stressed that rehabilitation strategies are personalized, accounting for the type of injury (acute vs. chronic) and job-specific tasks (e.g., ladder climbing, uneven terrain).

*“Every shoulder is different… they still need to be managed in a personalized way.”* ***PT07-Male.***

Physiotherapists reported conservative treatment approaches like targeted exercise programs in combination with manual therapy and various modalities depending on the type, stage, and severity of shoulder injury presented.

*“It depends on what stage of shoulder disorder, for example, in the acute phase when they are feeling high intense pain. I try to do relaxation exercises. Then we go further, starting the stretching exercises regarding the pattern that we are looking for. Going further, strengthening exercises might be added and at the end agility exercises, multi diagonal exercises and functional exercises will be the best option.”* ***PT10-Male.***

Similarly, other physiotherapists considered strategically simulating their treatment to the physical demands and practical end goal of the firefighter.

*“…I do think that the end stage rehabilitation, needs to be much more thorough than with a more sedentary or a population that has a less physically demanding job.”* ***PT05-Female.***

However, orthopedic surgeons reported another style of treatment based on their clinical expertise and experience managing FFs. This includes exploring both surgical and nonsurgical approaches like referring to physiotherapy prior to surgery and utilizing medications such as corticosteroid injections. However, if that fails, then a surgical style of management with physiotherapy post-surgery was indicated. Two orthopedic surgeons stated that:

*“…these (shoulder) problems are going to be treated non surgically initially and if the non-surgical treatment is not effective then we would consider sometimes diagnostic injections. Those treatments are usually non-surgical to start with, if that’s ineffective. Then surgical treatments like for our rotator cuff tear; we do a repair and in rare instances we do shoulder replacement surgery…”* ***OS09-Male.***

*“I think our acute management is typically an injection typically of a corticosteroid in the subacromial space with a concomitant physiotherapy program…if you know, imaging or our clinical scenario turns out to be a full thickness tear, then I tend to be leaning towards earlier surgery to get that fixed, you know soon and give them kind of maximum chance of a good outcome…”* ***OS08-Male.***

Furthermore, they also emphasized other factors including the current role or rank of the firefighter, age or year of experience and most importantly the presentation of shoulder injury determines the surgical technique utilized.

*“The number one procedure that I would perform would be an arthroscopic rotator cuff repair that would be for sure number one. And then after that probably labral repair, so, fixing anterior posterior labrum related to shoulder instability and that would be a slightly younger cohort. Biceps disease is very common, and it can be isolated or go along with some degree of rotator cuff disease. So often we do biceps tenodesis in this group where we move the location of the biceps attachment down to the proximal humerus.”* ***OS08-Male.***

Another surgeon also mentioned various surgical techniques based on the goal of the firefighter and the least desirable surgical technique due to the high physical demands of firefighting.

*“I think you know, rotator cuff repair is the most likely, …to get back to full strength and arthroscopic labral repair when they need a stable shoulder. I think they’re less likely to want to do a shoulder replacement. To treat arthritis they are more likely to put up with the symptoms and avoid replacement options for arthritis. Because they don’t want the permanent activity restrictions that are associated with an artificial shoulder.”* ***OS14-Male.***

Lastly, a surgeon noted that there are no surgeries uniquely specific to firefighters, but rotator cuff repairs, fracture fixation, and shoulder instability surgeries are common, and surgical decisions are based on injury type (acute vs. chronic).

*“I don’t know if there’s any (surgery) that would be specific for firefighters. Surgical treatments are common for fractures, instability of the shoulder... rotator cuff disease or... arthritis of the AC joints”* ***OS09-Male***

#### Subtheme 6.1: Incorporating self-management and treatment with job-specific and leisure activities.

Clinicians also underscored the need to incorporate the treatment into their occupational expectations, daily routines at home, training or leisure activities such as their gym routines outside of work.

*“Yes, I try to understand their job demands and their recreational demands, so their rank is important. So, knowing whether they’re a fire captain versus someone earlier in their career would dictate the physical nature and their expectations for function would be after treatment, but I do think it’s really important to dive into their personal situation and what they do outside of work, because the two are definitely synergistic in terms of the load on the shoulder.”* ***OS08-Male.***

Another clinician suggested providing personalized home exercise programs tailored to patient needs and environment or job demands.

“*Education should be easy to integrate into a regular routine... could maybe be implemented within job training sessions.”* ***PT05-Female***

Physiotherapists often recommended a concise, sustainable home exercise program post-discharge.

*“I ask my patients typically to maintain an exercise program semi regularly, so something to the tune of you know, two to three times per week, an exercise regimen for the shoulder that doesn’t last anywhere more than like 15 to 20 minutes.”****PT05-Female***.

They also highlighted the potential for incorporating such programs into job-related training contexts to increase long-term adherence and feasibility.

“*So, I mean, we always give them a home exercise program. That’s absolutely the key. And we do that on our own. … not a lot of online stuff that I would actually recommend. I just usually record and anything that I would kind of give them in terms of just what they need from an individualized assessment for what’s at home, what’s at their workplace in terms of exercise equipment to allow them to be able to understand, OK, this is how I’m going to manage this going forward in the long term versus the short term.”* ***PT01-Male***

#### Theme 7: (Re-)education on safe exercise training minimizes shoulder (re-)injuries.

Clinicians indicated that preventative education focusing on proper mechanics, postural awareness, and age-appropriate fitness training are crucial elements in minimizing WSDs. They emphasized transitioning from high-load workouts to low-load, high-repetition exercise routines and modifying job-related lifting and posture habits can reduce the risk for shoulder injuries.

*“…I think they do need to be smart about how they do dry land training, as their body ages, how they load and do dry land training needs to change. So, they can’t be doing the same workouts they did when they were 20 when you’re 50 and that we try to change the load volume from a high load, low repetition type workouts to high repetition, low load type workouts and avoid some of the position and exercises that I think may be overloading the rotator cuff specifically.”* ***OS08-Male.***

This was supported by a physiotherapist:

*“They’re in their 40s or 50s lifting like they’re in their 20s... that’s causing wear and tear. There’s a lot of education points on what overuse really is.”* ***PT01-Male.***

Further, a physiotherapist emphasized the importance of ergonomic awareness and conditioning that simulate firefighting duties:

*“…what we can do to make sure we don’t come back to this particular shoulder phenomenon anytime soon, relating it to their particular ergonomic positions. They move so quick they do so much. It’s difficult sometimes to set in some of these ergonomic suggestions just because of the environment. But if we’re able to relate to a pattern that’s going to be relevant, then we’re able to build into this, hopefully the subconscious a little bit of how we can structure that to their needs.”* ***PT04-Male.***

Clinicians also added that many FFs train with outdated or excessive exercise intensity, contributing to WSDs. Hence, they noted that preventive strategies hinge on education**,** particularly around biomechanics and rest to prevent overuse of shoulder structures.

“*…I counsel them on use of the shoulder and in terms of the riskier the positioning, the higher risk (for shoulder injuries) the activities would be. So, for example with rotator cuff repair, if they regain the ability to use the arm above shoulder level, and they regain their strength, theoretically they could go back to full duty, but there’s always a risk of recurrent tear, especially with heavier lifting above shoulder level. And so, the other aspect of it is that* *I tend to discourage heavier weightlifting in patients over 50 years of age because I’m always concerned about the risk of recurrent tear of the rotator cuff tendon.”* ***OS13-Male.***

A female clinician stressed the importance of shoulder health education and the negative impact of working through their shoulder pain instead of seeking professional help to optimize recovery during shoulder rehabilitation.

*“I think that’s where the education part is really important to let them know rates of tissue healing to remind them that just because it feels better doesn’t mean it’s 100% better. And to remind them that if things aren’t feeling well that they need to maybe come back and make some modifications to their rehab program in order to make sure that they can continue on, so not to delay or work through pain.”* ***PT03-Female.***

This was echoed by another clinician:

“*What I’ve found is usually when I start working with these firefighters, they have a history of shoulder issues that they kind of just worked through rather than going through a full rehab program.”* ***PT07-Male.***

#### Sub-Theme 7.1: Importance of tailored exercise delivery format for sustainable recovery.

There were varying opinions on the format of tailored exercise delivery to firefighters with WSDs. A group of clinicians discouraged passive formats (e.g., online videos, websites) and encouraged interactive seminars in fire halls, one-on-one sessions, and tailored in-person exercise training for practical engagement.

*“Well, I’m a big proponent of in-person. So, I mean, virtual is one thing, but there’s something to be said for being able to see people fully, see the front and back of them easily see what their reactions are. So, I’m all for in-person kinds of things. There’s a lot of research actually that shows that kind of small group activity-based training is pretty effective…there’s a benefit of being with your cohort. So, you know a couple of firefighters doing things together, they can see who’s doing what well, who isn’t, who might be struggling with something and help each other out. And I think that’s kind of the ideal environment in terms of providing both education and also correcting form and making sure everybody understands what we’re talking about in terms of making your shoulders healthy.”* ***PT03-Female***

These clinicians believe that prioritizing in-person education (e.g., safety meetings, peer-led demonstrations) results in better uptake and behavioral change. They added that the visible commitment of employers to workers’ health builds trust and participation in prevention of shoulder injuries.

“*I always like the in-person education piece... seminars in fire halls, in-service or one-on-one assessment…. Saying ‘watch this online video’... they may or may not watch it.”* ***PT01-Male.***

This was supported by another participant who indicated that:

*“I find that in-person sessions… are where you do get the greatest amount of benefit… there’s a better translation of knowledge.”* ***PT07-Male*.**

A second group of clinicians encouraged a virtual self-paced format of exercise delivery; they argued that it can accommodate the unique operational demands of firefighting.

***“…****virtual format is just as good as doing it in person…especially in a motivated population like firefighters.”* ***OS12-Male.***

Notably, they stated that this delivery format was more accessible and can be adapted to certain personal factors such as their current health or injury status.

*“I will say that I find the online is very helpful and accessible especially the websites I talked about, …that are credible. I will give firefighters journals around what the evidence is… what we do for this diagnosis, and what we did for their condition. So, I do a lot of online things”* ***PT02-Male.***

This group further expressed that intervention should be patient-centered and whatever format of exercise delivery including printouts from websites should be based on the preference of firefighters.

*“I think it depends on the person. So, is this person a visual learner? Is this person a learner that likes to do things? I think it’s always good to have a spectrum and I think you can have the same thing in different formats. Right. You can have a paper document and then have a website and you know, different things that you are able to deliver in different ways”* ***PT02-Male.***

A final group advocated for a hybrid format of exercise delivery including online and in-person exercise programs that can either be led by peers or physiotherapists.

*“I think hybrid would be the best and I think every firefighter or every station at least should have a physical therapy consultant with themselves regularly or on a regular basis to assess their posture and have some classes with them for education, and re-education and also they should have access to some resources, maybe online resources or video or images for their posture and exercises that they can do to prevent the problems.”* ***PT10-Male.***

This was supported by another clinician who stressed that:

*“…I think it’s probably a hybrid. I think that in person contact is much more appreciated because they can actually see what they’re doing right and wrong, which may not be as beneficial through a screen. But I also see the value (of virtual format), I sent a lot of home exercise programs virtually, and these programs have videos that they could refer to. So that helps as well.”* ***PT-06-Male***

#### Theme 8: Specialized functional assessment and outcome measures could enhance treatment outcomes.

Many physiotherapists reported using functional assessments and outcome measures to clinically assess and monitor recovery respectively. However, they claimed that performance-based functional assessments that mimic firefighter occupational context like pull-ups and grip strengths were preferred over traditional functional assessment or patient reported outcome measures (PROMs).

*“…I think that there are things we can look at in terms of you know, how many pull-ups they can do, all the way down through the arm into their grip strength. So, you know how well they can support themselves and support their body weight with their shoulders. So, there’s kind of how we measure, how long they can hang or how many pull-ups they could do. How long they can stabilize on one arm in a plank position for example, and then just general endurance kind of training things like how long they can do some kind of resisted band exercise very quickly over two or three minutes...”* ***PT03-Female.***

Another physiotherapist also mentioned that simulating work-specific tasks like overhead lifting and upper extremity weight bearing to mimic rescue operations during functional assessment was necessary for high-risk occupations like firefighting:

*“…You know, getting to simulating the task. And what they have to do, the physical training. Yeah. So again, I don’t do that for the average person and that’s why I said I need an hour to get through that. So, it’s beyond just range of motion exercises and strength. No, it’s your muscular balance, core stability. I go all out for firefighters, and I’ll do the same for my high performing individuals.”* ***PT02-Male.***

They stated that the most utilized PROMs is the Disabilities of the Arm, Shoulder and Hand (DASH). However, they preferred performance based functional outcomes that were more objective and job-relevant to guide their clinical decision making.

“…*Specific to firefighters, I do occasionally use the DASH, but besides that actually I also use the patient specific functional scale (PSFS)...”* ***PT05-Female.***

Other clinicians indicated that although traditional PROMs were mandatory for insurance boards and in making return to work (RTW) decisions but when combined with performance-based outcomes, it was more practical and helped track the recovery progress of FFs. However, there was no specialized performance-based outcome measure unique to FFs so there is always a need to adapt these traditional PROMs to the demands of firefighting.

*“…WSIB has a functional ability form, so that was a guideline by which we would be able to establish what their functional capabilities are. And that is if we’re looking at shoulders relating to lifting from floor to waist, waist to shoulder. So, I could add extra discussion there related to above shoulder work, how repetitive you’re using your shoulder above your head. How does your shoulder relate to other hand related activities such as grip dealing with vibrations or other of that phenomenon.”* ***PT04-Male.***

## Discussion

The findings of this study emphasized the need for specialized, occupation-specific approaches to prevention and management of WSDs among firefighters. Our findings showed the complexity of causative factors contributing to WSDs of FFs including environmental hazards, sociodemographic factors, organizational constraints, and exercise training deficiencies. Additionally, this study demonstrates that effective management of firefighter WSDs requires a comprehensive intervention that addresses the unique demands and constraints of firefighting operations.

Clinicians consistently underscored the unpredictable nature of firefighting as a major causative factor for WSDs. They reported that certain unavoidable environmental factors and routine exposures to occupational hazards during firefighting tasks increase their susceptibility to WSDs. Clinicians also highlighted that FFs often delay treatment and continue working through pain due to cultural norms surrounding toughness, camaraderie and duty. This behavioral pattern delays healing and exacerbates existing injuries coupled with the adverse scheduling of their shifts, often leading to fatigue, which increases their risk for WSDs [3]. This agrees with several studies emphasizing the dangerous and unpredictable nature of firefighting work and how it predisposes them to musculoskeletal injuries such as WSDs [6,22,23]. Our findings reinforce the need for both policy-level changes to accommodate cultural shifts within departments to normalize early reporting and functional work modifications to avoid stigmatization following a work-related injury.

Furthermore, the high physical demands of firefighting and associated training emerged as another causative factor that exacerbates WSDs [24]. Clinicians described firefighters’ routine tasks such as forcible entry, overhead lifting, ladder maneuvers, and dragging heavy equipment as inherently stressful to the shoulder complex, often leading to overuse injuries, acute trauma and cumulative strain. These findings support a Ghanian study by Kodom-Wiredu et al., [25] which showed a significant association between the high physical demands of firefighting tasks and WRMSDs such as WSDs. Several studies have also emphasized that these biomechanical loads are not isolated to fireground scenes but embedded within daily firefighter routines, including training drills and exercise training, increasing the likelihood of direct trauma, microtrauma or accumulation of musculoskeletal injuries like WSDs [26,27]. In fact many injured firefighter claims relate to training, not fireground injury [26].

Trauma-related incidents such as slips, trips, and falls were reported in our study as a prominent mechanism of shoulder injuries, particularly dislocations, labral tears, and fractures. These events were indicated to often occur during emergency responses on slippery surfaces, uneven terrain, or while carrying heavy loads. This is consistent with a qualitative study of North American firefighters by Osifeso et al., [3] which showed that unpredictable environmental conditions like lack of visibility during fireground emergencies was reported to cause traumatic shoulder injuries due to falls or slips. While other studies have widely recognized slips and falls as a hazard for musculoskeletal injuries among FFs [1,28,29]. This study provides clinician insight into the cohort of firefighters most predisposed to traumatic shoulder injuries, emphasizing how these cases differ from cumulative strain disorders and illustrating the mechanisms through which these injuries occur and present following acute, traumatic episodes in the workplace

Notably, clinicians in our study highlighted disparities in shoulder injury presentation based on sex and the experience level of FFs. Less experienced FFs were observed to suffer more acute traumatic injuries, potentially due to unfamiliarity with safe body mechanics under pressure and during training drills. In contrast, more experienced FFs tended to report chronic overuse syndromes of the shoulder. These findings support other literature on the increased risk of chronic work-related musculoskeletal disorders like WSDs among more experienced FFs [4,30,31]. Female FFs were also reported by clinicians to be underrepresented in their clinical caseloads when compared to their male counterparts. While a systematic review by Nazari et al., [2] reported that female FFs have a higher likelihood of experiencing multiple musculoskeletal disorders than their male counterparts, the large gender segregation of firefighters’ workforce, with 3–6% being female FFs, explains why they are often seen less by clinicians. These nuances suggest a need for more inclusive and tailored prevention and treatment strategies that consider career stage and sex-specific risk factors among FFs.

Clinicians across disciplines repeatedly cited the absence of structured, occupation-specific training programs focused on shoulder health. While general physical training was embedded in firefighter routines, many clinicians noted a disconnect between traditional fitness regimes and the neuromuscular demands of the job. This gap was identified as a missed opportunity for proactive prevention, and the lack of simulated exercise training routines has been emphasized in various studies to predispose one to WRMSDs such as WSDs [27,32]. For instance, a study by Smith et al., [33] recommended the need for a targeted fitness program that mimics the job of firefighting. Our study showed that without a targeted strengthening and mobility routines that simulate firefighting scenarios, FFs are left vulnerable to imbalances and poor movement patterns that contribute to WSDs.

Timely and accurate diagnosis was unanimously viewed by clinicians as a cornerstone of effective treatment. Delays in seeking care or in receiving appropriate diagnostic imaging and referrals were reported to often cause progression from an initially manageable injury into chronic or complex WSDs. For instance, a recent qualitative study by Osifeso et al., [9] emphasized how timely diagnosis when managing FFs with WSDs could enhance successful treatment outcomes and reduced recovery times. Likewise, various studies have shown that early intervention facilitates the use of non-surgical strategies in less severe shoulder injuries before surgical pathways become necessary [34,35], which aligns with current best practices of shoulder injury musculoskeletal care [36].

Moreso, some clinicians expressed caution in over-reliance on diagnostic tools without adequate clinical context. While MRI and ultrasound were deemed valuable for structural or anatomical confirmation [34,37], clinicians underscored the importance of skilled clinical assessment, including detailed history taking and functional evaluations. This hybrid diagnostic approach ensured that treatment plans were both evidence-based and contextually relevant to the firefighter’s occupational demands, physiological, and psychosocial context. Our findings complement current clinical practice guidelines for the nonsurgical management of shoulder injuries, which encourage an initial thorough clinical assessment while considering physiological, occupational and psychosocial factors before recommending diagnostic imaging [36].

There was strong consensus among clinicians that a one-size-fits-all approach is ineffective for managing shoulder injuries among FFs. Rather a customized multimodal approach was recommended, which integrates manual therapy, functional exercise, modalities and ergonomic education. This agrees with a systematic review and meta-analysis by Aguliar Gracia et al., [38] which showed that although therapeutic exercise is the cornerstone for treating less severe cases of shoulder disorders, however, integrating educational interventions, manual therapy and other modalities like laser therapy showed substantial benefits. Our findings also reported that surgical management is usually indicated when nonsurgical treatment fails or when a shoulder injury interferes with firefighting duties or daily routines. This was supported by a systematic review of clinical practice guidelines, which recommended a surgical approach when there is significant functional limitation or persistent pain for 3–6 months following nonsurgical management of WSDs [39]. Likewise, several studies have established that the effectiveness of this approach is attributed to personalizing multimodal care based on patient needs [36] and occupational demands [40,41].

Successful shoulder rehabilitation in our study was linked to the integration of self-management strategies and the incorporation of exercises that reflected occupational and recreational activities. Clinicians emphasized the importance of aligning rehab goals with firefighter-specific duties and personal lifestyle to enhance compliance and return-to-duty outcomes. For example, a randomized control trial conducted in Brazil by Santello et al., [42] showed that teaching home exercise programs to patients with shoulder disorders helped improve function and reduce pain in participants. This reinforces the evidence that self-management strategies with formal treatment interventions represent a component of successful management of WSDs [36,43], especially for FFs with WSDs [9]. Our findings highlight the importance of empowering firefighters with tools and knowledge to actively participate in their recovery while accommodating the irregular schedules and unpredictable demands inherent in their occupation and personal lives. However, firefighting simulation, wearable sensors, and work-site visits were rarely mentioned by physical therapists and given the nature of such a high-demand occupation, this might be considered a gap. Since firefighters report they have difficulty accessing therapy that is tailored to the nature of their work, it is important to acknowledge what was “not said” and how that might improve the quality and specificity of shoulder rehabilitation for firefighters. Firefighters are at elevated risk of occupational cancers and occupational stress injuries, and these were rarely mentioned as potential mediators of health that might impact assessment or management of firefighters with shoulder injury.

Several studies have shown the substantial benefit of re-education of appropriate and safe exercise programs in the prevention and management of shoulder injuries [39,44,45]. Our findings demonstrate that effective injury prevention requires firefighters to understand what exercises to perform and how to perform them safely while carrying out their firefighting duties. Many clinicians expressed concern that firefighters often returned to gym-based training or exercise routines that replicated the movement patterns that caused their shoulder injuries and some report that exercise behaviors like poor form or emphasizing maximum weight lifted might increase injury risk. This finding agrees with a US study by Poplin et al., [27] which showed that about 32% of occupational injuries reported by firefighters were linked to unsafe training exercise routines. This unsafe exercise educational gap perpetuates a cycle of reinjury and reinforces the need for proactive, evidence-informed exercise training frameworks that are safe, accessible and culturally accepted within fire services.

There was a lack of consensus regarding which exercise training delivery format, whether in-person coaching, digital modules, or peer-led sessions, was most efficient. While some clinicians called for the integration of virtual self-paced formats with feedback mechanisms to enhance long-term engagement among firefighters, other clinicians highlighted in-person peer or clinician-led sessions for sustained recovery from WSDs. This mixed narrative is supported by a randomized controlled trial comparing the effectiveness of digital and in-person intervention delivery formats for patients with shoulder disorders, the results showed similar improvements in treatment outcomes [46]. Despite the mixed narrative regarding the type of exercise delivery format, most participants agreed that exercise programs, irrespective of the delivery format, should accommodate their varying fitness levels, operational demands and the diverse learning preferences of firefighters.

Lastly, performance-based functional outcomes such as range of motion assessments, grip strength benchmarks, and task-specific simulations were most preferred by clinicians. However, it was noted that incorporating performance-based functional outcomes and standardized functional PROMs like the DASH were mandatory for making RTW decisions for firefighters. Clinicians emphasized that traditional PROMs, while valuable for tracking general recovery parameters, were lacking in capturing specific functional demands that define successful return to firefighting duties. Few mentioned concerns about the DASH not being approximately targeted for firefighters (ceiling effect) which raises concerns about how these are used in decision-making. Macdermid et al., [47] demonstrated that firefighters’ self-reported work limitations on generic PROM demonstrate ceiling effects and thus do not correlate to their actual performance of firefighting tasks. Hence, the development and implementation of firefighter-specific functional assessment and outcomes are needed for comprehensive assessments, while “work-hardening” and gradual return-to work programs are needed to ensure a safe and sustainable return to duty [10].

## Limitations

Several limitations should be considered when interpreting the findings from our study. Our study focused primarily on Canadian healthcare contexts, which may limit transferability to other international firefighting and healthcare systems with different organizational structures and treatment approaches, although our findings aligned with studies from other countries. Also, our findings are derived from self-reported accounts, which may not fully capture actual behaviors or outcomes due to the subjective interpretation and the possibility of recall bias in clinicians’ responses.

## Implications for practice and policy

Fire departments and health services should co-develop evidence-informed shoulder rehabilitation frameworks that integrate clinical benchmarks, functional capacity data, and firefighter feedback. These frameworks should account for task variability, environmental exposures, and psychosocial readiness to prevent premature RTW causing shoulder re-injury. Likewise, fire departments should establish policies ensuring rapid access to specialized healthcare services, while insurance boards should provide flexible coverage for extended rehabilitation protocols when necessary to avoid extensive delays which could affect the recovery of firefighters with WSDs.

## Future recommendations

Future research should prioritize developing and validating firefighter-specific performance-based shoulder and work assessment tools that capture unique occupational demands of FFs. Strategies for tailoring exercise to job performance requirements, prevention of injury especially in non-emergency contexts like exercise and training drills, and clinical practice guidelines that address high-demand occupations might improve the quality of rehabilitation experienced by firefighters. Considering firefighter health more holistically including age, job tenure and gender influences on shoulder disorder susceptibility, reflects broader knowledge gaps in literature that require further investigation. Lastly, clinicians should pursue specialized training in firefighter occupational health and establish multidisciplinary teams knowledgeable about firefighting context. This is recommended to encourage the development of partnerships with fire departments to facilitate targeted workplace accommodation and ensure a comprehensive understanding of their operational demands during management and RTW decisions.

## Conclusion

Our findings reveal that clinicians with experience managing WSDs among firefighters recognize the interplay of occupational, individual, and organizational factors that require specialized clinical understanding and targeted management approaches. The reported disparities in shoulder injury patterns based on work experience and gender further emphasize the need for individualized assessment and occupation-centered treatment approaches that account for diverse risk profiles among firefighters. Gaps in the risks of firefighter non-musculoskeletal disorders, and the use of firefighter-specific outcome measures, sensorized performance monitoring, firefighter task analysis and use of fire-specific graded training were notably not discussed. Effective shoulder disorder management in this population requires enhanced collaboration between healthcare providers, fire departments, and firefighters to ensure therapists support a proactive prevention and early injury response approach to shoulder injury that might extend the probability of a long and healthy career as a firefighter.

## Supporting information

S1 File Physiotherapist interview guide. (DOCX)

S2 File Surgeon interview guide. (DOCX)
