# Supplementary material for: “Every shoulder is different”: A qualitative study of clinicians’ insights on the causative factors and strategies of managing work-related shoulder disorders among firefighters
Source: PLoS One. 2026 May 21;21(5):e0348934. doi: 10.1371/journal.pone.0348934 (PMC13193412; doi:10.1371/journal.pone.0348934)
Supplement: S1 File — (DOCX) [file pone.0348934.s001.docx]

**Title: Perspectives of Firefighters and Clinicians on the Causes and Management of Work-Related Shoulder Disorders: An Interpretive Descriptive Approach**

**Semi-Structured Interview Guide for Physiotherapist Clinicians**

**Introductory Preamble**

Welcome to this interview designed for clinicians who treat firefighters with work-related shoulder injuries.

The goal of this interview is to gain insight into the experiences and challenges that clinicians who work with this firefighters’ face, and to better understand how to support them in their efforts to promote firefighters' health and wellness.

Through this interview, we hope to gain a better understanding of the approaches and strategies used by clinicians when treating firefighters with shoulder injuries especially when managing pain, facilitating rehabilitation, and preventing further injury.

Do I have your permission or consent to record the interview?

During your practice, have you ever managed firefighters with shoulder injuries?

**Focused Question on the Causes of Work-Related Shoulder Injuries among Firefighters**

1. What are the common shoulder injuries or disorders among firefighters that you have encountered in your practice

- Probe- Tell me about your experience managing these shoulder problems among firefighters?

1. Are firefighters the same or different from other patients with shoulder injuries?
   - Probe - Any distinct pattern or characteristics in shoulder injuries among firefighters that is different from other population?
2. Are there any work-related tasks or specific challenges firefighters face that increase the risk of shoulder injuries?
3. What other common factors do you think contribute to shoulder problems in firefighters?
   - Anatomic
   - Training
   - Exercise, personal lifestyle, other side jobs
4. How do you think work-related shoulder injuries affect the job of firefighting?

**Focused Questions on the Management of Work-Related Shoulder Injuries**

**Diagnosis**

1. Can you discuss how important early detection and timely diagnosis is in managing shoulder disorders among firefighters?
2. What diagnostic tools and techniques do you find most valuable for assessing shoulder conditions in this population?
   - Probe - Do you do anything differently during diagnosis when your patient is a firefighter?
   - Probe - Are there any diagnostic challenges in detecting shoulder injury in this population
3. Can you discuss how important early detection and timely diagnosis is in managing shoulder disorders among firefighters?
4. How do you investigate the specific causative factors that are relevant for firefighters with respect to their shoulder injury?
   - Probe - What causative factors do you typically explore?

**Prevention**

1. From your perspective, what preventative measures or recommendations can be implemented for firefighters to reduce the risk of shoulder injury or re-injury
2. How do you educate or re-educate firefighters on self-care practices or ergonomic adjustments to minimize the risk of shoulder injuries
   - What format might be best to implement these self-care practices or ergonomic adjustments to prevent WSDs among firefighters?
3. How do you teach safe exercises, lifting or carrying to firefighters to prevent or minimize the risk of shoulder injuries during work-related activities?
   - Do you think firefighters are aware of this safe exercise, lifting and carrying practices or not?

**Treatment**

1. Can you talk about shoulder injury rehabilitation that you have employed for firefighters with WSDs?
   - How do you ensure that firefighters do no over exert themselves during exercise routines or rehabilitation programs
2. How do you address firefighters' concerns about re-injury or future shoulder problems?
3. What treatment modalities or strategies have been shown to be effective in managing shoulder injuries among firefighters
   - Probe – Pre or post surgery?
4. Do you employ any specialized rehabilitation or PT programs for firefighters with shoulder injuries?
   - Are there any specific outcome measures specific to them functional or not?
5. What is the long-term outlook for firefighters with shoulder disorders?
   - Probe – Strategies employed to promote ongoing shoulder health throughout their careers?
6. How do you relate with other interprofessional services when managing WSDs amongst firefighters?

**Recovery/Return to work**

1. What is the recovery rate of firefighters that you have managed?
2. How do you ensure that firefighters with shoulder injuries are compliant with treatments or rehab?
3. How do you work with firefighters to set realistic rehabilitation goals post shoulder injuries?
4. Are there any specific challenges or considerations in helping firefighters safely return to work or their physically demanding roles?
5. How do plan for return to work?
   - Probe -Could you outline a typical return to work process for firefighters? recovering from shoulder disorder?
   - Probe - How do you plan for them to return to their physically demanding expectations and roles at work?
6. How do you make sure your firefighters have access to the resources they need to manage their shoulder injury after rehab?
   - Probe – What are the common follow up PT management services mostly required? Examples such as pain management.

**Closing**

We understand that clinicians' work is complex, and we appreciate your willingness to share your insights with us. Your input will be invaluable in assisting us to develop strategies and protocols to assist clinicians working with firefighters.

Thank you for taking the time and for taking part in this interview. I will now end the interview.
