# Supplementary material for: “Every shoulder is different”: A qualitative study of clinicians’ insights on the causative factors and strategies of managing work-related shoulder disorders among firefighters
Source: PLoS One. 2026 May 21;21(5):e0348934. doi: 10.1371/journal.pone.0348934 (PMC13193412; doi:10.1371/journal.pone.0348934)
Supplement: S2 File — (DOCX) [file pone.0348934.s002.docx]

**Semi-Structured Interview Guide for Surgeons**

**Introductory Preamble**

Welcome to this interview designed for clinicians who treat firefighters with work-related shoulder injuries.

The goal of this interview is to gain insight into the experiences and challenges that clinicians who work with this firefighters face, and to better understand how to support them in their efforts to promote firefighters' health and wellness.

Through this interview, we hope to gain a better understanding of the approaches and strategies used by clinicians when treating firefighters with shoulder injuries especially when managing pain, facilitating rehabilitation, and preventing further injury.

Do I have your permission or consent to record the interview?

During the course of your practice, have you ever managed firefighters with shoulder injuries?

**Focused Question on the Causes of Work-Related Shoulder Injuries among Firefighters**

1. What are the common shoulder injuries or disorders among firefighters that you have encountered in your practice

- Probe- Tell me about your experience managing these shoulder problems?

1. Are firefighters the same or different from other patients with shoulder injuries?
   - Do you do anything differently during diagnosis when your patient is a firefighter?
   - Any distinct pattern or characteristics in shoulder injuries among firefighters that is different from other population?
2. What common factors do you think contribute to shoulder problems in firefighters?
   - Anatomic
   - Work related.
   - Training
   - Others e.g., Exercise, personal lifestyle, other side jobs
3. How do you investigate the specific factors that are relevant for firefighters with respect to their shoulder injury?

- What factors apart from their occupation do you typically explore?

1. How do you think work-related shoulder injuries affect the job of firefighting?

**Focused Questions on the Management of Work-Related Shoulder Injuries**

**Diagnosis**

1. Can you discuss how important early detection and timely diagnosis is in managing shoulder disorders among firefighters?
   - Why? Briefly discuss.
   - What diagnostic tools and techniques do you find most valuable for assessing shoulder conditions in this population?
   - Are there any diagnostic challenges in detecting shoulder injury in this population

**Treatment**

1. Can you discuss the shoulder surgeries that are commonly indicated for firefighters?
2. What treatment modalities or strategies have been shown to be effective in managing shoulder injuries among firefighters
3. Are there surgical interventions that are frequently required for firefighters?
   - Probe: What are the considerations for surgical management in this population?
4. Referral procedure prior to getting in your care
   - Are you the first point of contact
5. What are the barriers and facilitators to Management of shoulder injuries

**Return to work**

1. How do plan for return to work?
   - Probe - How do you plan for them to return to their physically demanding expectations
2. How do you make sure your firefighters have access to the resources they need to manage their shoulder injury?
   - Probe – What are the common follow up management services mostly required? Examples such as physical therapy, occupational therapy or pain management?

**Recovery**

1. What is the recovery rate of firefighters that you have managed?
2. How do you address firefighters' concerns about re-injury or future shoulder problems?
   - Probe-Mental concerns, financial concerns, and physical concerns?
3. Re-injury and how you managed it?
4. Prevention recommendations?
5. Safe exercise format for firefighters?
6. Take home messages regarding shoulder injuries among firefighters

We understand that clinicians' work is complex and difficult, and we appreciate your willingness to share your insights with us. Your input will be invaluable in assisting us in developing strategies and protocols to assist clinicians working with firefighters.

Thank you for taking the time and for taking part in this interview. I will now end the interview.
